# The State of the Inguinal Glands in Primary Syphilis

**Published:** 1901-04-20

**Authors:** 


### THE STATE OF THE INGUINAL GLANDS IN PRIMARY SYPHILIS.

In the diagnosis of primary syphilis the condition of the inguinal glands is a matter on which much stress is always laid, and properly so. Mr. Arthur Cooper has, however, lately pointed out that the diagnostic value of such glandular enlargement depends more on the circumstances of the particular case than on what may by chance be discovered at any particular time. In the examination of any case in which there is a lesion within the area drained by the lymphatics which go to the inguinal glands the first thing is, he says, not to assume without due inquiry that any glandular enlargement which may be present is the direct consequence of that lesion. Different persons vary greatly as regards the susceptibility of their glands. Tuberculous subjects seem to be the most liable to have the lymphatic glands affected on slight provocation, but many others are easily vulnerable in this respect; and just as some children get enlarged cervical glands from irritation on the scalp while others in the same circumstances escape, so with the inguinal glands, which in many men and boys are found to be enlarged to an extent which may be of considerable importance in regard to the subject in hand. In some such cases there has been adenitis in connection with some previous morbid process, but in many the patient is either quite ignorant of the presence of the enlarged glands, or if he knows of them attributes them to some such cause as running, jumping, or what not. In some cases the surgeon can detect no cause for the condition, but Mr. Cooper thinks that in most of them some form of mild but long-continued irritation is present—most frequently a condition of balano-posthitis, such as may be due to a prepuce worn habitually forward and insufficiently cleansed. The important point is that a man who is apparently healthy and without any history of venereal or other disease to account for it, may have a degree of indolent enlargement of the inguinal glands which of itself is indistinguishable from that which is found in some cases of syphilis. In typical cases the diagnosis is easy, but all cases are not typical, so that if a patient comes for advice for enlarged glands, accompanied by some form of lesion of the genital organs, the first question which we have to decide is whether the enlarged glands are due to that lesion or not, and if we know nothing of the previous condition of the glands this question cannot be decided at once by the glands themselves. Not only however may the inguinal glands be enlarged from causes other than venereal, but in many cases of primary syphilis the enlargement may be very slight, and in some no enlargement at all can be felt. Most of the cases in which the latter condition has occurred have either been very fat or have been in the habit of wearing a truss, the pressure of which seems sometimes to prevent enlargement taking place. Some cases have also been recorded of absence of enlargement in connection with phagedenic primary sore. Again, even although enlargement may be venereal it may not be syphilitic. There may be mixed infections: early enlargement from a soft chancre may hide a later enlargement indicating syphilis, or a sore may have been so irritated with caustic as to have set up a glandular enlargement suggestive of syphilis, or along with a suspicious sore there may be gonorrhœa or other urethral irritation to which the glandular enlargement may be due.

